# IoT-Enabled Fog-Based Secure Aggregation in Smart Grids Supporting Data Analytics

**DOI:** 10.3390/s25196240

**Published:** 2025-10-08

**Authors:** Hayat Mohammad Khan, Farhana Jabeen, Abid Khan, Muhammad Waqar, Ajung Kim

**Affiliations:** 1 Department of Computer Science, COMSATS University, Islamabad 45550, Pakistan; hayathk@hotmail.com; 2 College of Science and Engineering, University of Derby, Derby DE22 1GB, UK; a.khan3@derby.ac.uk; 3 Department of Computer Science, Faculty of Arts and Sciences, Edge Hill University, Ormskirk L39 4QP, UK; m.waqar@uos.ac.uk; 4 Department of Optical Engineering, Sejong University, Seoul 05006, Republic of Korea

**Keywords:** fog computing, IoTs, smart grid, privacy preservation, fault-tolerance, homomorphic encryption, data analytics, BGN, statistical analysis, ANOVA

## Abstract

The Internet of Things (IoT) has transformed multiple industries, providing significant potential for automation, efficiency, and enhanced decision-making. The incorporation of IoT and data analytics in smart grid represents a groundbreaking opportunity for the energy sector, delivering substantial advantages in efficiency, sustainability, and customer empowerment. This integration enables smart grids to autonomously monitor energy flows and adjust to fluctuations in energy demand and supply in a flexible and real-time fashion. Statistical analytics, as a fundamental component of data analytics, provides the necessary tools and techniques to uncover patterns, trends, and insights within datasets. Nevertheless, it is crucial to address privacy and security issues to fully maximize the potential of data analytics in smart grids. This paper makes several significant contributions to the literature on secure, privacy-aware aggregation schemes in smart grids. First, we introduce a Fog-enabled Secure Data Analytics Operations (FESDAO) scheme which offers a distributed architecture incorporating robust security features such as secure aggregation, authentication, fault tolerance and resilience against insider threats. The scheme achieves privacy during data aggregation through a modified Boneh-Goh-Nissim cryptographic scheme along with other mechanisms. Second, FESDAO also supports statistical analytics on metering data at the cloud control center and fog node levels. FESDAO ensures reliable aggregation and accurate data analytical results, even in scenarios where smart meters fail to report data, thereby preserving both analytical operation computation accuracy and latency. We further provide comprehensive security analyses to demonstrate that the proposed approach effectively supports data privacy, source authentication, fault tolerance, and resilience against false data injection and replay attacks. Lastly, we offer thorough performance evaluations to illustrate the efficiency of the suggested scheme in comparison to current state-of-the-art schemes, considering encryption, computation, aggregation, decryption, and communication costs. Moreover, a detailed security analysis has been conducted to verify the scheme’s resistance against insider collusion attacks, replay attack, and false data injection (FDI) attack.

## 1. Introduction

**Figure 1:**
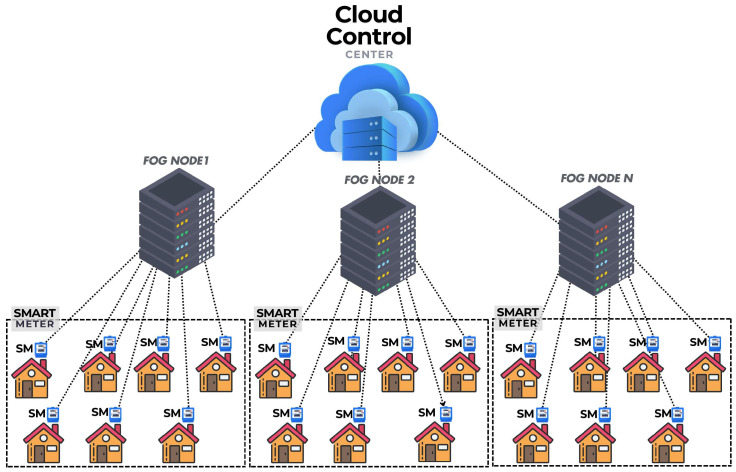
Architecture of a typical Fog-enabled Smart Grid Infrastructure.

Smart Grid (SG) combines electric and communication network components to improve the efficient and consistent delivery of environmentally friendly and cost-effective electricity. It optimizes the economic, environmental, operational, and reliability aspects of electrical service provision. Currently, the operational and commercial elements of energy infrastructures are becoming more and more dependent on the integration of the Internet of Things (IoT) into the SG [1]. Additionally, this integration gives the SG network the ability to communicate in real time and in both directions with consumers, utilities, and infrastructures. It includes software components for controlling and monitoring power usage at the production and consumption ends [2]. A typical SG includes appliances, a smart meter (SM), Gateway/aggregator (GW), and a control center (CC). In traditional cloud-based systems, both public and private data from SGs are usually stored and processed at the cloud control center (CCC) [3]. Electricity usage data is obtained from customer premises at regular intervals and sent to the CCC. This information is extremely beneficial to SG operations management. The use of analytics for electricity usage data facilitates the identification of consumer trends, seasonal variations, and consumption patterns. These insights are instrumental in load forecasting, resource availability, pricing models, churn management, demand response, and the optimization of SG operations. Within SGs, data analytics and statistical analysis play a crucial role in extracting meaningful insights. Statistical analytics, as a core component of data analytics, employs various methods to uncover patterns and trends within usage datasets, enabling businesses and organizations to make informed decisions and advance big data analytics and predictive modeling in SG environments. However, the handling of customers’ private data raises privacy concerns. Without proper security and privacy measures, sensitive customer information, including living habits, types of equipment used, and daily activities, may be exposed to potential attackers. Consequently, it is essential to ensure that customer metering data is collected securely and shared only with authorized parties. However, these issues, along with storage and computing challenges, can be mitigated by employing a secure fog-based SG architecture [4]. Fog computing [5] transfers storage and computation overhead from the CCC to nearby-user edge terminals managed by fog nodes (FNs), complementing cloud computing by enabling direct interaction between IoT devices and applications, leading to a significant reduction in bandwidth requirements compared to cloud computing. A typical fog-based SG architecture that supports aggregation is illustrated in Figure 1. The fog node (FN) has the capability to perform aggregation operations on SM data and temporarily store it locally for a brief period. As it is close to the end user’s location, it improves overall communication. To safeguard SMs data from malicious users, it is encrypted at the SM level and then aggregated at the FN, which subsequently transmits the aggregated values to the CCC. The CCC is responsible for decrypting the received aggregated values [6].

Given the emphasis on privacy and security in SG operations, conducting data analytics at the fog level closer to the network edge where data is generated provides potential benefits in tackling security and privacy issues. Fog computing provides solutions for reliability, latency, and processing time, thus bolstering the integrity of SGs and addressing apprehensions regarding the security and privacy of customer data. Handling faulty SMs missing data during data aggregation is also crucial. If some SMs fail to report their consumption data, the data aggregation process may be disrupted. Consequently, unless all SMs submit their data to the aggregator, the aggregation process cannot be finalized. Therefore, fault tolerance must be a supported feature of data aggregation. Nevertheless, there are several limitations to current secure fog-enabled data aggregation approaches for SGs [6,7,8,9,10,11] that only focus on calculating total consumption at CCC. While such approaches facilitate the calculation of current load, enabling more sophisticated analyses such as average consumption, peak consumption, load forecasting, and state estimation require support for more advanced computational functions. Performing complex statistical operations on encrypted data is also challenging, as the actual SMs data needs not to be revealed and keeping a low overhead in terms of computation and storage. There exists work related to aggregation schemes for non-fog-enabled SGs that do support operations like average and variance [2,12]. The scheme [2] leverages the Boneh-Goh-Nissim (BGN) cryptosystem, allowing the gateway to perform aggregation of encrypted data without decrypting individual user data. The scheme incorporated differential privacy protection by adding geometric noise at the gateway to aggregated encrypted data to protect against differential attacks. The scheme allows the CCC to support multifunctional statistical computations (e.g., averages, variances, ANOVA). Although effective in safeguarding privacy, the scheme is vulnerable to inaccuracies when smart meters fail, as missing data leads to erroneous decryption results. It also lacks mechanisms for data integrity and authentication. Additional mechanisms are needed to prevent data tampering or injection attacks. The approach relies heavily on gateways for aggregation, but these devices are resource-constrained, limiting scalability in dense SG environments. The scheme assumes the gateway and control center are trusted entities. If either is malicious or compromised internally, privacy guarantees may be weakened. Geometric noise addition for differential privacy introduces errors in the aggregated results. There is still a trade-off between privacy level and accuracy. The scheme [12] employs additive Paillier homomorphic encryption and identity-based signatures to ensure secure aggregation, message integrity, and protection against replay and impersonation attacks. While effective for basic sums, Paillier supports only limited ciphertext operations and is inefficient for higher-order statistical computations such as variance or covariance and ANOVA. This scheme also used GW as a data aggregator and suffered from congestion and scalability due to increased load. Furthermore, the absence of fault tolerance means that missing data from smart meters delays decryption and increases communication overhead, with gateways posing potential bottlenecks and single points of failure. The scheme assumes that aggregators and smart meters behave honestly and does not provide mechanisms for detecting or mitigating malicious behavior, such as false data injection or collusion attacks. No explicit handling of malicious GW (aggregator) or smart meters is provided.

The key contributions of this paper are summarized as follows:

- The Fog-enabled Secure Data Analytics Operations (FESDAO) scheme offers a distributed architecture that supports real-time processing at the fog level while incorporating robust security features such as secure aggregation, authentication, and resilience against insider threats. Privacy and confidentiality of SM data are ensured through modified homomorphic Boneh-Goh-Nissim (mBGN) cryptosystem and other mechanisms. Only FN and CCC can decrypt aggregated data. Moreover, our scheme is resilient against replay attacks and false data injection (FDI) attacks, and it ensures security even in the presence of potentially malicious SM, FN and CCC. In addition, the scheme is resistant against collusive attacks.
- ⁠Unlike current state-of-the-art schemes that support statistical analytics on encrypted data only at the CCC after decryption [2,12], our FESDAO scheme enables secure analytical operations such as average, variance, and ANOVA functions directly on encrypted data at both the FN and CCC levels. This capability allows for real-time insights into energy consumption patterns, facilitating the design of dynamic tariff plans. Customers can make informed decisions by selecting optimal tariff plans that align with their consumption patterns. Furthermore, the FESDAO scheme is fault-tolerant, guaranteeing that statistical operation evaluations remain unaffected and latency is not compromised, even in the presence of malfunctioning smart meters It enhances reliability in real-world deployments with intermittent failures.
- The FESDAO scheme has been analyzed for security, statistical function support on encrypted data, computational, and communication overheads. The results are then compared to the current state-of-the-art schemes [2,12], particularly in encryption, aggregation, decryption, and computation cost efficiency. Moreover, a detailed security analysis has been conducted to verify the scheme’s resistance against insider collusion attacks, replay attacks, and false data injection (FDI) attacks

The remainder of this paper is organized as follows: In Section 2, related work is presented. In Section 3, the preliminaries of the BGN cryptosystem, MAC, and privacy-preserving statistical operations (Average, Variance, and ANOVA) are presented. In Section 4, the system model and security goals are discussed. Section 5 presents the proposed fault-tolerant secure data aggregation scheme. In Section 6, we provide a privacy analysis of the proposed scheme. The performance evaluation of the scheme is discussed in Section 7. Finally, Section 8 concludes this work.

## 2. Related Work

This section explores existing data aggregation systems that use privacy features to improve data privacy and reliability. Shi et al. [13] proposed the DG-APED system, which detects errors and classifies users based on activity time. In this technique, noise is added at the SM level and aggregated at the group level. Differential privacy is achieved using binomial distribution. Bao et al. [7] proposed an AES-based approach with differential privacy utilizing a Laplacian distribution. This technique also allows for data integrity and fault tolerance. Borges et al. [14] proposed an in-network data aggregation technique that protects privacy via homomorphic commitment and digital signatures. While this approach reduces encryption costs at the SM level, it has a significant decryption cost at the data receiver end. Erkin et al. [15] investigated the geographical aggregation of SM measurements using cryptographic techniques to ensure privacy. Furthermore, their system enables the temporal aggregation of multiple usage readings for specific SMs.

Tonyali et al. [16] were the first to use Fully Homomorphic Encryption (FHE) and Secure Multi-Party Computation (SMPC) to protect client usage data. This method enables hierarchical, safe aggregation while preserving consumer privacy. Abdallah et al. [17] suggested a technique using a lattice-based cryptosystem. In this strategy, data from client appliances is collected and sent to the appropriate SMs, who encrypt it using an NTRU-based cryptosystem before sending it to the CCC. Thoma et al. [18] created an SMPC-based, homomorphic encryption solution for SG load management. This approach protects consumer privacy by using homomorphic encryption. Lyu et al. [8] proposed a differentially private aggregation scheme with built-in fault tolerance, employing OTP-based additive homomorphic encryption. In this approach, plaintext data is combined with key streams using modular addition, while privacy protection for metering data is ensured through Gaussian noise distribution. However, the scheme is vulnerable to false data injection (FDI) attacks. In addition, it introduces communication delays and requires an additional communication round in the presence of failed meters.

Yang et al. [9] suggested an anomaly detection technique that incorporates dynamic grouping and data re-encryption using the ElGamal encryption scheme. However, their scheme exposes the identities of SMs, failing to protect consumer privacy and anonymity during data aggregation. Saleem et al. [6] propose a privacy-preserving data aggregation technique for fog-based SGs that uses modified Paillier homomorphic encryption to secure metering data and message authentication codes (MAC) for integrity verification. However, their fault-tolerance mechanism relies on dummy values, leading to inaccurate aggregation results. Additionally, their scheme does not ensure data confidentiality against a potentially malicious control center, posing security risks in adversarial environments. Khan et al. [11] a solution that combines the BGN cryptosystem and the Elliptic Curve Digital Signature Algorithm (ECDSA). Their system offers secure aggregation and privacy of encrypted data, as well as fault tolerance for malfunctioning smart meters.

Chen et al. [12] suggested a technique based on the Paillier cryptosystem. This approach enables suppliers to compute the total metering data usage while preserving anonymity. It also allows variance analysis and ANOVA by combining numerous data reports into a single message. Chen et al. [2] proposed the Multi-Functional Data Aggregation (MuDA) approach, which uses homomorphic encryption to guarantee privacy-preserving calculations on SM data. Their approach supports statistical functions, including summation, average, and one-way ANOVA, on encrypted data at the CCC levels. However, the scheme does not include mechanisms for addressing faulty smart meter data during the aggregation process, which could compromise the accuracy and reliability of the results obtained. Zhao et al. [19] developed a fog-based SG architecture that allows for statistical computations on consumption data. Zhang et al. [20] introduced a lightweight and resilient strategy for multidimensional data aggregation in the Internet of Things (IoT). The Chinese Remainder Theorem (CRT) is used in their strategy to compress SM multidimensional data into 1-D data. Encryption is accomplished using mask values and symmetric keys, and batch verification cuts computational complexity in half. This technique somehow raises aggregation costs at FNs. Pang et al. [21] presented a Boolean/Arithmetic secret-sharing technique that allows for secure transmission of electricity usage data to the CCC and FN. Their approach uses Boolean shares to detect realistic consumption limits while maintaining privacy, anonymity, and resilience against both honest-but-curious and malevolent attackers. Zhang et al. [22] proposed a fault-tolerant multidimensional data aggregation strategy based on the Elliptic Curve-ElGamal model. This technique provides privacy and accuracy during data aggregation even in the presence of defective SMs. It avoids collusion assaults and ensures accurate electricity bill creation, but actual implementation details are not provided. Song et al. [23] created a fault-tolerant data aggregation approach that encodes multidimensional data and weights with the Chinese Remainder Theorem. Their privacy-preserving solution operates without the need for a trusted third party, utilizing the Paillier homomorphic encryption system and a secure key negotiation mechanism. Fault tolerance is obtained by an enhanced Shamir secret sharing mechanism. Zhang et al. [24] proposes a blockchain-based multidimensional data aggregation scheme for SGs that integrates advanced cryptographic techniques to achieve decentralization, privacy preservation, identity authentication, and forward secrecy. The scheme supports multidimensional aggregation and fault tolerance, enabling smart meters to report multiple data types per message and allowing flexible users to join/leave operations through dynamically verifiable secret sharing. A Raft-based leader election mechanism is used to select aggregation nodes; however, its reliability depends on the assumption that a majority of nodes are honest and online, which may not hold in adversarial or unstable environments. While blockchain enhances integrity and auditability, it also introduces latency and storage overhead, raising concerns for large-scale deployment. Performance results show reduced computation and communication costs compared to related approaches, highlighting the scheme’s practical efficiency for SG applications.

## 3. Preliminaries

This section offers a summary of the cryptographic primitives utilized in this paper. We briefly describe the working of mBGN cryptosystem, MAC, and statistical functions execution on aggregated data. For details on these topics, we refer the reader to [22,23,24,25].

### 3.1. BGN Cryptosystem

The BGN cryptosystem [26] is a homomorphic encryption scheme that enables efficient and privacy-preserving computation on encrypted data [7]. Unlike Paillier, which supports only addition over ciphertexts, BGN additionally supports the multiplication of two encrypted values. This makes it particularly suitable for operations that require both addition and multiplication, such as variance, covariance, dot products, and other statistical computations beyond simple averaging. It is also applicable to machine learning tasks (e.g., distance calculation, clustering, neural networks), quadratic or higher-degree analytics, and privacy-preserving range queries and comparisons. Notably, BGN was the first scheme to support both addition and multiplication of ciphertexts while maintaining constant ciphertext size [27].

### 3.2. Key Generation

Select large primes $q_{1},q_{2}$ such that $n=q_{1}q_{2}$. $G,G_{1}$ are the two multiplicative groups of order n with a bilinear pairing $e:(G\times G)\to G_{1}$. Select two random generators g,u from group G and set $h=u^{q_{2}}$. The private and public keys are generated as $pk=(n,G,G_{1},e,g,h)$ and $sk=q_{1}$. In the proposed scheme FESDAO, we use a 1024-bit key, generated from two 512-bit primes $q_{1}$ and, $q_{2}$. Although the BGN cryptosystem supports larger key sizes (e.g., 2048 and 4096 bits), a 1024-bit key is selected to account for the computational and storage limitations of sensor devices.

#### 3.2.1. Encryption

Providing a message m and selecting a random number ${r\in Z}_{N}^{*}$, encrypted text can be calculated as $g^{m}h^{r}\in G$.

#### 3.2.2. Decryption

Using the symmetric key sk = $q_{1}$ and the encrypted text C∈G, compute $C^{q1}=(g^{m}{h^{r})}^{q_{1}}$ and $C^{q_{1}}=(g^{q_{1}}{)}^{m}$. Recover the message m using a discrete logarithm.

### 3.3. MAC Algorithm

The MAC calculation is a symmetric-key cryptographic technique used to ensure message integrity and verify the source. It confirms that the received message comes from a legitimate source and prevents any unauthorized alterations during transmission [6,28]. Bob, as the sender party, computes MAC as a function of the message m and the shared secret key k, i.e., $M=MAC_{k}(m)$.

Bob sends both the message m and the authentication tag M to the receiving party, Alice. Upon receiving the message, Alice calculates the MAC for it and compares it with the MAC she has. If both MACs match, Alice accepts the message m, confirming that it is from a legitimate source and has not been tampered with during transmission. In the above communication, if MAC at the receiving end will produce incorrect results it means the message m was altered in transit. The mechanism described above ensures message authentication and integrity.

### 3.4. Privacy-Preserving Aggregation and Statistical Functions Calculation

The FN can aggregate encrypted metering data through homomorphic encryption for i = 1,2,3 …n user’s data in a privacy-preserving manner. The proposed scheme supports commonly used statistical aggregation functions, namely average, variance, and ANOVA at FN and CCC levels. It can be easily extended to other statistical functions.

#### 3.4.1. Average Calculation

When there is a request to calculate the average of consumption data at the FN or CCC levels, it is calculated through a function $Avg=\frac{1}{n}\;\sum_{i=i}^{n}m_{i}$. The FN aggregates the encrypted data for all meters under its jurisdiction, decrypts it, and divides it by the number of SMs.

#### 3.4.2. Variance Calculation

When there is a request to calculate the variance of usage data at the FN or CCC levels, it is calculated through a function $\sum_{i=1}^{n}{\left(m_{i}-\bar{m}\right)}^{2}$. Here $\bar{m}$ denotes the average value of all metering data. The encrypted data for all meters in format $\prod_{i=1}^{n}c_{i}.\prod_{i=1}^{n}c_{i}$ is decrypted, and variance is obtained. 

#### 3.4.3. ANOVA Calculation

ANOVA, or Analysis of Variance, is a parametric statistical method developed by R.A. Fisher for comparing datasets [29]. It is particularly useful for assessing the means and variances among more than two groups. In the context of SG applications, ANOVA can be employed to determine whether a specific usage package significantly affects customers’ electricity consumption data.

In this paper, to illustrate the computation of ANOVA, we used a running example involving three distinct electricity plans. The ANOVA analysis was performed on sample data collected from smart meters associated with a fog node, corresponding to three different electricity tariffs labeled A, B, and C.

In the ANOVA function, the source of variance between groups and within groups is used to calculate the F-value through the F-Test expression. The F-value can be utilized to ascertain whether a specific tariff scheme influences customers’ electricity consumption. Steps for ANOVA calculation are as follows:

Suppose we have K groups (i.e., tariff plans).

Let each Group j has $n_{j}$ observations: $x_{1j}$, $x_{2j}$, … $x_{nj}$

Total number of observations is $N=\;\sum_{j=1}^{k}n_{j}$

Calculating Group mean: $\bar{x_{j}}$ = $\frac{1}{n_{j}}$ $\sum_{i=1}^{n_{j}}x_{ij}$

Calculate Overall Mean: $\bar{x_{0}}$ = $\frac{1}{N}$ $\sum_{J=1}^{K}\sum_{i=1}^{n_{j}}x_{ij}$

Compute Sum of Squares Between Groups (SSB): $\sum_{j=1}^{k}n_{j}{\left({\bar{x}}_{j}-\bar{x_{0}}\right)}^{2}$

Compute Sum of Squares Within Groups (SSW): $\sum_{j=1}^{k}\sum_{i=1}^{n_{j}}{\left({\bar{x}}_{ij}-{\bar{x}}_{j}\right)}^{2}$

Compute Total Variation (SST): SSB + SSW

Calculate Mean Squares (MSB): $\frac{SSB}{\left(K-1\right)}$

Mean square within groups (MSW): $\frac{SSW}{\left(N-K\right)}$

Calculate F-Statistic: MSB/MSW

Degrees of freedom: ${df}_{B}$ is equal to number of groups minus 1, ${df}_{W}$ is total number of observations minus number of groups

Hypothesis Testing: Compare the calculated F with the critical value from the F-distribution with $\left(K-1,N-K\right)$ degrees of freedom at the chosen significance level (e.g., α = 0.05). If $F>F_{critical}$, reject the null hypothesis $H_{0}$ (that all group means are equal).

The ANOVA analysis was conducted on sample data for three different electricity tariffs, A, B, and C from SMs connected to FN. Sample values are given in Table 1. To perform secure analytical operations, count ($n_{j}$), ∑$x_{j}$ and ∑$x_{j}^{2}$ operations are performed directly on encrypted data using the mBGN cryptosystem. Decryption activity is only performed at FN/CCC. No individual SM data is exposed during aggregation or other operations required for ANOVA.

**ANOVA from aggregates (no raw data required):**
Between-groups SS (SSB) = 8.416022
Within-groups SS (SSW) = 19.935330
Total SS (SST) = 28.351352
Degrees of freedom: ${df}_{B}$ = 2, ${df}_{W}$ = 69
Mean Squares: MSB = 4.208011, MSW = 0.288918
F-statistic: F = 14.564734
*p*-value ≈ $5\times 10^{-6}$ (significant at α = 0.05)
Reject $H_{0}$ (equal group means).
**Effect size (eta-squared):** η^2^ = 0.296847 (~29.7% of variance explained by group).

The corresponding SSB is 8.416022 and SSW is 19.935330, yielding an F-statistic of 8.305 with degrees of freedom (2, 69). The associated *p*-value (≈$5\times 10^{-6}$) is less than 0.05, leading to the rejection of the null hypothesis of equal means.

## 4. System Model and Security Goals

This section covers the discussion of the system model, attacker model, and security goals.

### 4.1. System Model

**Figure 2:**
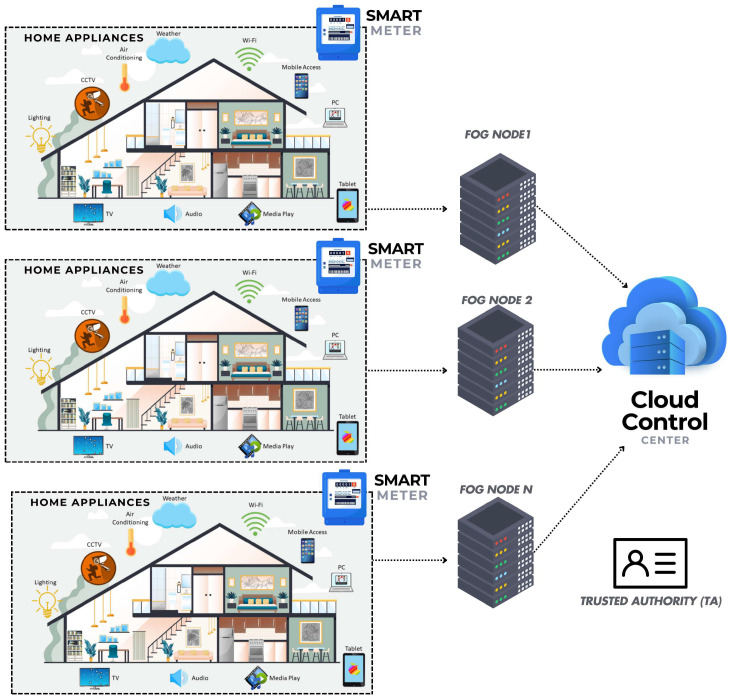
System model.

The system model comprises the following components, as illustrated in Figure 2:

**Trusted Authority (TA):** The TA oversees the registration of SG entities and the generation of their keys. Any changes to entity details must be updated in the TA database, and the TA contacts SM vendors to reinstate SM configurations if necessary.

**CCC:** The CCC is a highly trusted entity in SG responsible for collecting metering data through FN. It performs analytical operations on usage data and manages SG operations, including demand-response, forecasting, and billing.

**FN:** The FN is responsible for collecting metering data from the SMs, aggregating it, and transmitting it to the CCC. It also performs analytical operations at the FN level when necessary.

**SM:** SMs are advanced metering infrastructure (AMI) devices that monitor the flow of electricity from the grid to residential homes. They also submit household usage data to FN at regular intervals. If households act as prosumers if they sell their excess electricity to suppliers and charge them.

**Home Appliances (AP):** Each home contains various appliances such as fridges, microwaves, washing machines, and TVs, all of which consume electricity and submit their usage data to the SM installed in the respective household. The SM then submits the aggregated usage data to FN on behalf of these appliances.

### 4.2. Attacker Model

In the proposed system, FN and CCC are honest but curious. Despite being unable to access individual customer data due to security measures, they are curious about viewing private customer information. The mBGN cryptosystem is utilized to encrypt the usage data, leveraging its homomorphic properties to prevent FN or any other involved parties from reading SM data in plain text. The source of SM data is authenticated at FN through MAC, and the FN collects and aggregates data from all connected SMs, which are considered tamper-resistant devices. The proposed attacker model includes the following scenarios:

1. An adversary eavesdropping on the communication between SM and CCC.
2. External adversaries can manipulate messages from SM to FN or from FN to CCC.
3. The adversary goals may include knowing the aggregated and individual SM readings
4. SMs do not transmit data due to malfunction.
5. An adversary compromising CCC.
6. An adversary initiating the following attacks:
  (a) Replay attack
  (b) FDI attack
  (c) Message modification attack
  (d) Unauthorized access
7. Assumptions
  (a) CCC and FN are honest but curious
  (b) CCC and FN may collude
  (c) Residential users/SMs are considered honest
  (d) Communication channel is not secure

### 4.3. Security Goals

The proposed scheme accomplishes the following security objectives:

**Privacy:** In the event of an attacker intercepting communication packets over an insecure channel, it is infeasible to extract private customer information. The FN cannot access encrypted customer data, as it lacks a decryption mechanism unless necessary. The CCC can decrypt aggregated data from the FN, but not individual customer data. Even in the event of a compromise, only current aggregated data can be leaked, while individual customer data remains secure.

**Fault Tolerance:** The aggregation process will persist even in the presence of malfunctioning SMs. The CCC will decrypt aggregated data from the FN for both functioning and non-functioning SMs.

**Data Integrity:** Any modification of data packets from SM to FN during transit will result in their rejection. Only data packets from authorized entities are accepted.

Any attempts by an adversary to send previously stored packets will be thwarted using timestamps.

## 5. Proposed Scheme

This section outlines the proposed FESDAO scheme. The primary stakeholders are the Application Provider (AP), Smart Meters (SMs), Fog Nodes (FNs), the Cloud Control Center (CCC), and the Trusted Authority (TA). The TA is responsible for generating cryptographic keys for SMs, FNs, and the CCC using the modified BGN (mBGN) cryptosystem. These keys are used for encryption and decryption operations. In addition, the TA generates secret keys for HMAC, which are used by SMs, FNs, and the CCC for message authentication code (MAC) generation and verification.

The scheme supports statistical function computations at both the FN and CCC levels. At the SM level, home appliance data is collected, encrypted using mBGN, and transmitted to the FN. At the FN, the encrypted data is aggregated in a privacy-preserving manner. Depending on the computation requirements, the FN can either perform statistical calculations (e.g., average, variance, ANOVA) on the aggregated ciphertexts or forward the aggregated data to the CCC. At the CCC, decryption and additional statistical analysis can be performed on the aggregated results.

**Figure 3:**
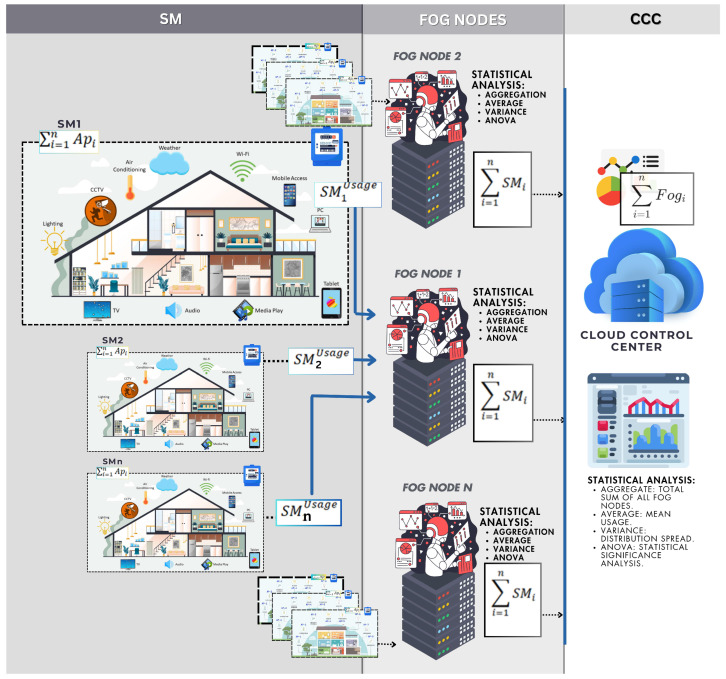
Analytics calculation at FN and CCC level.

Figure 3 illustrates the flow of metering data and the execution of statistical functions within the proposed scheme. The framework ensures compliance with data protection regulations by safeguarding customer data through robust cryptographic and security mechanisms.

The operation of the FESDAO scheme is defined through five core algorithms: (i) Key Generation, (ii) Encryption and MAC Generation at the SM, (iii) MAC Verification and Fault-Tolerant Secure Data Aggregation at the FN, (iv) Decryption of Aggregated Data at the FN, and (v) Statistical Function Computation at the FN. The notation and symbols used throughout the scheme are summarized in Table 2.

**Table 2:** List of symbols.

Symbol	Description
SM	Smart Meter
FN	Fog Node
CCC	Cloud Control Center
*q*_1_	Private Key of BGN
(n,G,G1,e,g,h)	Public Key of BGN
$s_{0}$	Secret parameter of CCC
$s_{i}$	Secret parameter of SM/User
$f_{i}$	Secret parameter of FN
$m_{i}$	Message text
R	Random number
$t_{i}$ or t	The timestamp for a particular time interval
$S_{req}$	Statistical Function calculation request form FN
${MAC}_{{sm}_{i}}$	MAC tag generated on $c_{i}$ at ${sm}_{i}$
${MAC}_{{fn}_{i}}$	MAC tag generated on received $c_{i}$ at FN
$c_{i}$	Ciphertext at SM (metering data as m)
$c_{s}$	Ciphertext at SM (metering data as m*m)
buf	Buffer storage for the current reading of $sm_{i}$
SMinfo	Data structure contains ${SM}_{id}$, $p_{k}^{sm}$, and *Flag*
*Flag*	Status field, 0 for failed meter, 1 for working meter
$C_{fn}$	The aggregated ciphertext of all SMs at ${FN}_{i}$
${Agg}_{val}$	Aggregated value at CCC
$M_{sqrsu}$	Sum of square metering data
$H\left(c_{i}\right)$	Hash function using SHA-256
M/$M_{sum}$	Sum of all SMs consumption data
$C_{d}$	All fog nodes’ aggregated value
${sm}_{w}$	Working SM
${sm}_{f}$	Failed SM

### 5.1. Key Generation

This task focuses on generating the cryptographic keys for the mBGN cryptosystem and the secret parameters required by SMs and the CCC to enable fault-tolerant decryption. In the mBGN scheme, the public key is used for homomorphic encryption, while the private key enables homomorphic decryption.

The TA begins by selecting two large prime numbers $q_{1}$ and $q_{2}$ and computes n = $q_{1}.q_{2}$. Using bilinear (pairing) $e:(G\;\times \;G)\;\to G_{1}$ and random group generators g and u, the TA computes $h=u^{q_{2}}$. The TA then publishes the public key $\left(n,G,G_{1},e,g,h\right)$ and securely retains the private key $q_{1}$ for the mBGN cryptosystem. To support fault-tolerant decryption, the TA further selects n random numbers $s_{i}$ from $Z_{N}^{*}$ using a random generation function, where i=1,2,3…,n and assign each $s_{i}$ to ${SM}_{i}$. Additionally, TA also selects $s_{0}\;\mathrm{and}\;f_{i}$ from $Z_{N}^{*}$ and assigns to the CCC and FN in such a manner that the below conditions hold. Equation (1) can be used if decryption at the CCC level is required, while Equation (2) will be used if decryption is required at the FN level.

(1) $$s_{0}.(s_{1}+s_{2}+\ldots s_{n})=1\;mod\;q_{1}$$

(2) $$f_{i}.(s_{1}+s_{2}+\ldots s_{n})=1\;mod\;q_{1}$$

This inverse relationship enables the system to recover the original aggregated ciphertext (or, equivalently, the required exponent) by combining the partial results from the smart meters and then applying a single combining coefficient. In essence, the combining coefficient counteracts the effect of the aggregated shares, allowing the aggregator to obtain the exact ciphertext or exponent necessary to complete the decryption process.

For MAC generation and verification, TA, as discussed in [30], generates a private key sk for SM and FN, respectively. Once the key generation activity is completed, the TA will only be required to add or remove SMs if they join or leave the SG network. The steps for this step are given in Algorithm 1.
**Algorithm 1.** Key generation algorithm.  1.**Procedure** Key Generation  2.**Input:** Large Prime numbers $q_{1},q_{2},n=q_{1}q_{2}$  3.**Output:** Public Key $pk=\left(n,G,G_{1},e,g,h\right),Private\;Key\;sk=q_{1}$, Secret Parameters $s_{i},$ $s_{0}$, $f_{i},$ and $s_{i}$. Private key $s_{k}$ for MAC generation  4.Let $G,G_{1}$ be two multiplicative groups of order n with a bilinear pairing e:(G × $G)\to G_{1}$  5.Pick two random generators g,u $\overset{R}{\leftarrow }$ G and set $h=u^{q_{2}}$  6.Then h is a random generator of the subgroup G order $q_{1}$  7.${{Generate\;s}_{i}\in Z}_{N}^{*}$, i = 1,2, 3, …n  8.Generate ${s_{0}\in Z}_{N}^{*}$, i = 1,2, 3, …n  9.Generate ${f_{i}\in Z}_{N}^{*}$, i = 1,2, 3, …n  10.Compute ${f_{i}\in Z}_{N}^{*}$ s.t $f_{i}.\sum_{i=1}^{n}s_{i}$ $=1\;mod\;q_{1}$  11.Compute ${s_{0}\in Z}_{N}^{*}$ s.t $s_{0}.\sum_{i=1}^{n}s_{i}$ $=1\;mod\;q_{1}$  12.Generate $s_{k}$ for MAC generation and verification  13.**Return** $(s_{0}$, $s_{i}$, $s_{k}$, $f_{i}$)  14.**end procedure**

### 5.2. Encryption and MAC Generation at SM

This task is responsible for encryption and MAC generation at the smart meter. Each $SM_{i}$, encrypts its data $m_{i}$ using its secret key $s_{i}$, producing ciphertext $c_{i}$. A MAC ${MAC}_{{sm}_{i}}$ on ciphertext $c_{i}$ is generated using the secret key $s_{k}$. The encryption and MAC operations are performed through Equations (3) and (4). In these equations $t_{i}$ represents the timestamp corresponding to a specific time slot. If there is a requirement for statistical function calculation, the FN issues a request $S_{req}$ to each SM. When $S_{req}$ value is Yes, both $c_{i}$ and $c_{s}$ are submitted to enable the computation of statistical functions such as average, variance, and ANOVA functions. Finally, the ciphertext $c_{i},c_{s}$ along ${MAC}_{{sm}_{i}}$ are submitted to the FN.

(3) $$c_{i}=E(m_{i})=g^{{rtm}_{i}}\;h^{rts_{i}f_{i}}$$

(4) $${MAC}_{{sm}_{i}}=s_{k}(H(c_{i}||t_{i}))$$

The steps for this task are given in Algorithm 2.

**Algorithm 2.** Encryption and MAC generation algorithm.
  1.**Procedure** Encryption and MAC Generation  2.**Input:** Message $m_{i}$, time $t_{i}$, $S_{Req}$, ${SM}_{i}$  3.**Output:** Cipher $c_{i},$ MAC ${MAC}_{{sm}_{i}},$ Cipher $c_{s}$  4.$c_{i}$ = E($m_{i})$ ${=\;g}^{{rtm}_{i}}$ $h^{rts_{i}f_{i}}$  5.If $S_{Req}$ = Yes  then  6.$c_{s}$ = E($m_{i}$ *×* $m_{i})$ ${=\;g}^{{rtm_{i}m}_{i}}$ $h^{rts_{i}f_{i}}$  7.end if  8.${MAC}_{{sm}_{i}}$ = $S_{k}^{sm}$ (H($c_{i})$ || $t_{i}$)  9.**Return** $({MAC}_{{sm}_{i}}$, $c_{i}$, ${SM}_{i}$, $c_{s}$)  10.
**end procedure**



### 5.3. Fault Tolerant Secure Aggregation of Data at FN

In this task, the FN maintains a multi-column list SMinfo consisting of two fields: ${\;SM}_{id}$ and *Flag*. At the beginning of each data collection round the *Flag* column value for every SM is initialized to 0. When data $c_{i}$ is received from a particular SM, the FN retrieves the corresponding ${SM}_{id}$ from SMinfo and calculates its MAC $\left({MAC}_{{fn}_{i}}\right)$ for verification purposes. If the MAC is valid, the FN updates the *Flag* value for that SM to 1 and stores a copy of the metering data in a local buffer buf, indexes by ${SM}_{id}$.

The FN then aggregates the ciphertext values received from all connected SMs under its domain. The FN verifies the count of all SMs in his jurisdiction based on the flag value in SMinfo list. If some SMs fail to send their data (where *flag = 0*), the FN substitutes their missing readings with the most recently stored valid readings from the buffer.

The aggregation operation is performed using Equations (5a) and (5b). If all SMs successfully transmit their data, Equation (5a) is used; otherwise, Equation (5b) is applied. It is assumed that at least one valid reading for every SM exists in the buffer buf.

(5a) $$C_{fn}=\prod_{i=1}^{n}c_{i}$$

$$if\;SM_{faulty}\;then$$

(5b) $$C_{fn}=\prod_{i=1}^{w}c_{i}+\prod_{i=1}^{f}buf(c_{i})$$

The FN aggregates the encrypted metering data for both working and failed SMs. For working SMs’, the data corresponding to its current timestamp t, where for failed SMs, the buffered values with timestamp $t_{b}$ are used:

$$C_{fn}={\left(\prod_{i=1}^{w}c_{i}\right)}^{t^{-1}}+\;{\left(\prod_{i=1}^{f}c_{i}\right)}^{t_{b}^{-1}}$$

$$C_{fn}={\left(g^{rt\sum_{i}^{w}m_{i}}\;h^{r{tf}_{i}\sum_{i}^{w}s_{i}}\right)}^{t^{-1}}+{\left(g^{rt_{b}\sum_{i}^{f}m_{i}}\;h^{r{t_{b}f}_{i}\sum_{i}^{f}s_{i}}\right)}^{t_{b}^{-1}}$$

Assuming t ≈ $t_{b}$, we obtain:

$$C_{fn}={\left(g^{rt\sum_{i}^{k}m_{i}}\;h^{r{tf}_{i}\sum_{i}^{k}s_{i}}\right)}^{t^{-1}}$$

Taking t common from *g* and *h*:

$$C_{fn}={\left(g^{r\sum_{i}^{k}m_{i}}\;h^{rf_{i}\sum_{i}^{k}s_{i}}\;\right)}^{tt^{-1}}$$

$Since\;f_{i}$.$\sum_{i}^{n}s_{i}=1\;(mode\;q_{1}$), the final aggregated ciphertext is:

(5c) $$C_{fn}=g^{r\sum_{i}^{n}m_{i}}\;h^{r}$$

To support statistical function calculations (e.g., variance and ANOVA), the FN also performs squaring operations on ciphertexts, producing $C^{{fn}_{sqragg1}}$ for available SMs, and $C^{{fn}_{sqragg2}}$ for faulty SMs. Once aggregations are complete, the FN generates its own MAC on aggregated ciphertext $C_{fn}$ as defined in Equation (5d).

(5d) $${MAC}_{fn}=s_{k}^{fn}\left(H(C_{fn})\;||\;t_{i\;}\right)$$

The steps for this task are given in Algorithm 3.
**Algorithm 3.** Aggregation and MAC verification algorithm at FN.  1.**Procedure** Aggregation and MAC Verification at FN  2.**Input:** ${MAC}_{{sm}_{i}}$**,**$c_{i}$**,**$c_{s}$**,**${SM}_{i}$  3.**Output:**$C^{{fn}_{agg}}$**,**$C^{{fn}_{sqragg}}$  4.${/*\;SM}_{info}$ is Multi field data structure. It has fields ${SM}_{i}$, and Flag. */  5./* In start of data collection round, Flag default value set to 0 */  6./* Step 7 to 14 repeat for all SMs who submitted their data in specific data collection round*/  7.${MAC}_{{fn}_{i}}$ = $S_{k}$ (H ($c_{i})$ || $t_{i}$)  8.If ${MAC}_{{fn}_{i}}$ = ${MAC}_{{sm}_{i}}$ then  9.$\;MAC\;is\;verified\;and\;c_{i}\;is\;accepted$  10. Based on ${SM}_{i}$, ${SM}_{info}.Flag$ = 1  11.$\;{buf}_{i}$ = (${SM}_{i},$ $c_{i}$, $t_{i})$  12.Else  13. MAC is rejected and $c_{i}$ is discarded  14.end if  15./* ${sm}_{w}$ contains SM whose flag in ${SM}_{info}$ 1.   16.For $i=0;{i<sm}_{w}$; i++ do  17.$\;C^{{fn}_{agg1}}$ = $\prod_{i=1}^{w}c_{i}$  18.$\;C^{{fn}_{sqragg1}}$ = $\prod_{i=1}^{w}c_{s}$  19./* ${sm}_{f}$ contains SM whole flag in ${SM}_{info}$ is 0, i.e., they are failed meters */  20.For $i=0;i<{sm}_{f}$; i++ do  21.$\;C^{{fn}_{agg2}}$ = $\prod_{i=1}^{f}{buf}_{i}$ (${sm}_{f}$, $c_{i}$, $t_{i}$)  22.$\;C^{{fn}_{sqragg2}}$ = $\prod_{i=1}^{f}{buf}_{i}$ (${sm}_{f},c_{i},t_{i}$) * ${buff}_{i}$ (${sm}_{f},c_{i},t_{i}$)  23.${C^{{fn}_{agg}}=C}^{{fn}_{agg1}}$ + $C^{{fn}_{agg2}}$  24.${C^{{fn}_{sqragg}}=C}^{{fn}_{sqragg1}}$ + $C^{{fn}_{sqragg2}}$  25.**Return** $\left(C^{{fn}_{agg}},C^{{fn}_{sqragg}}\right)$  26.**end procedure**

### 5.4. MAC Verification and Fault-Tolerance Secure Data Aggregation at FN

In the proposed scheme, the FN performs decryption operations to compute aggregated usage data and to support the execution of statistical functions such as average consumption, variance, and ANOVA. The decryption process is carried out using Equations (6) and (7).

(6) $$C_{d}=g^{r\sum_{i}^{n}m_{i}}\;h^{r}$$

Respective FN computes:

(7) $$C_{d}h^{-r}=\;g^{r\sum_{i}^{n}m_{i}}$$

The FN then computes the discrete logarithm of $g^{r\sum_{i}^{n}m_{i}}$ to recover aggregated values $M_{sum}$ and M_sqrsum_. The step is accomplished using Pollard’s lambda method [28], as defined in Equations (8) and (9).

(8) $$M_{sum}=\sum_{i}^{n}m_{i}$$

(9) $$M_{sqrsum}={\left(\sum_{i}^{n}m_{i\;}\right)}^{2}$$

The steps for this task are given in Algorithm 4.
**Algorithm 4.** Decryption and MAC verification at FN algorithm.  1.**Procedure** Decryption at FN  2.**Input:** $C^{{fn}_{agg}},\;C^{{fn}_{sqragg}}$  3.**Output:** $M,\;M_{sqrsum}$  4.$C_{d}$ = $C^{{fn}_{agg}}$  5.$C_{d}$ = $g^{r\sum_{i=1}^{n}m_{i}}$ $h^{r}$  6.$C_{d}$ = $\frac{g^{r\sum_{i=1}^{n}m_{i}}}{{h-}^{r}}$  7.$C_{d}$ $h^{r}$= $g^{r\sum_{i=1}^{n}m_{i}}$  8.FN through discrete log get $M=\;g^{r\sum_{i=1}^{n}m_{i}}$  9.FN through discrete log get $M_{sqrsum}=\;g^{{\left(r\sum_{i=1}^{n}m_{i}\right)}^{2}}$  10.**Return** ($M,\;M_{sqrsum}$)  11.**end procedure**

### 5.5. Statistical Functions Calculation at FN

In this task, the Fog Node (FN) performs the computation of aggregated consumption, average consumption, variance, and ANOVA functions. For this task, the SM must submit the data in the required format, as discussed in Algorithm 2. Once the aggregated ciphertexts (both in their standard and squared forms) are received, they are decrypted using Pollard’s lambda method. The FN then derives the average and variance using Equations (10) and (11).

$$M_{avg}=\frac{1}{n}M_{sum}$$

(10) $$M_{avg}=\frac{1}{n}\sum_{i=1}^{n}m_{i}$$

$$M_{var}=\frac{1}{n}\;M_{sumsqr}-\frac{1}{n^{2}}\;M_{sqrsum}$$

(11) $$M_{var}=\frac{1}{n}\;\sum_{i=1}^{n}m_{i}^{2}-{\left(\frac{\sum_{i=1}^{n}m_{i}}{n}\right)}^{2}$$

In addition to variance, the scheme also supports the execution of the ANOVA test to evaluate the impact of different tariff plans on electricity consumption behavior. Let us assume there are s different tariff plans (e.g., P1, P2, P3). Customers may choose one of these plans depending on their pricing preferences. Once the FN receives aggregated consumption data for each tariff plan, it calculates the following values:

It calculates the sum of squares of all data through $A_{1}=$ $\sum_{j=1}^{s}\sum_{i=1}^{n}m_{ij}^{2}$, calculates Sum of squares of aggregated data within each tariff plan using $A_{2\;}$ $=\;\sum_{j=1}^{s}{\left(\sum_{i=1}^{n}m_{ij}\right)}^{2}$ and Square of the sum of all data across plans using $A_{3\;}={\left(\sum_{j=1}^{s}\sum_{i=1}^{n}m_{ij}\right)}^{2}.$ As per ANOVA, the sum of squares between (s different tariff plans) is

$$\;SS_{B\;}=\sum_{j=1}^{s}\sum_{i=1}^{n}m_{ij}^{2}-\frac{1}{n}\sum_{j=1}^{s}{\left(\sum_{i=1}^{n}m_{ij}\right)}^{2}$$

(12) $$SS_{B\;}=A_{1}-\frac{1}{n}\;A_{2}\;$$

and the sum of squares between within groups is

$$SS_{W\;}=\frac{1}{n}\sum_{j=1}^{s}{\left(\sum_{i=1}^{n}m_{ij}\right)}^{2}-\frac{1}{ns}{\left(\sum_{j=1}^{s}\sum_{i=1}^{n}m_{ij}\right)}^{2}$$

(13) $$\;SS_{W\;}=\frac{1}{n}\;A_{2}-\frac{1}{ns}\;A_{3}$$

Finally, the FN performs the F-Test to evaluate whether the differences among tariff plan groups are statistically significant:

(14) F= SSB(s−1)SSw(n−s) 

Here s−1 denotes the degree of freedom between group and n−s within the groups. The computed F value (with the numerator n−1 and denominator n−s) is compared with a critical threshold at a chosen significance level (commonly 5%). If the calculated F value is below the threshold, this indicates that the group means differ significantly, suggesting that at least one tariff plan influences customer electricity usage. Conversely, if the F-value exceeds the threshold, it implies no significant difference among group means, indicating that the different tariff strategies do not have a measurable effect on electricity consumption.

The steps for this task are given in Algorithm 5.
**Algorithm 5.** Statistical functions calculation algorithm.  1.**Procedure** Statistical Functions  2.**Input:** ${Req}_{avg}$, ${Req}_{var}$, $n_{Sm}$  3.**Output:** Avg,Var  4.Variance can be calculated using $\sum_{i=1}^{n}{\left(m_{i}-\bar{m}\right)}^{2}$  5.$c_{i}$ = $\sum_{i=1}^{n}m_{i}$  6.$c_{i}^{sqr}$ = $\sum_{i=1}^{n}m_{i}^{2}$  7.$c_{fogi}$ = $\prod_{i=1}^{n}c_{i}$  8.$c_{fogisqr}$ = $\prod_{i=1}^{n}c_{i}^{sqr}$  9.Var= $\frac{c_{fogisqr}}{n_{Sm}}$ − ${\left(\frac{c_{fogi}}{n_{Sm}}\right)}^{2}$  10.Var= $\frac{1}{n_{Sm}}\sum_{i=1}^{n}m_{i}^{2}$ − ${\left(\frac{\sum_{i=1}^{n}m_{i}}{n_{Sm}}\right)}^{2}$  11.Var= $\frac{1}{w_{Sm}}\sum_{i=1}^{n}m_{i}^{2}$ − $\frac{1}{w_{sm}^{2}}$ ${\left(\sum_{i=1}^{n}m_{i}\right)}^{2}$  12.Avg= CSmaggnSm  13.**Return** $\left(Avg,Var\right)$  14.**end procedure**

**Figure 4:**
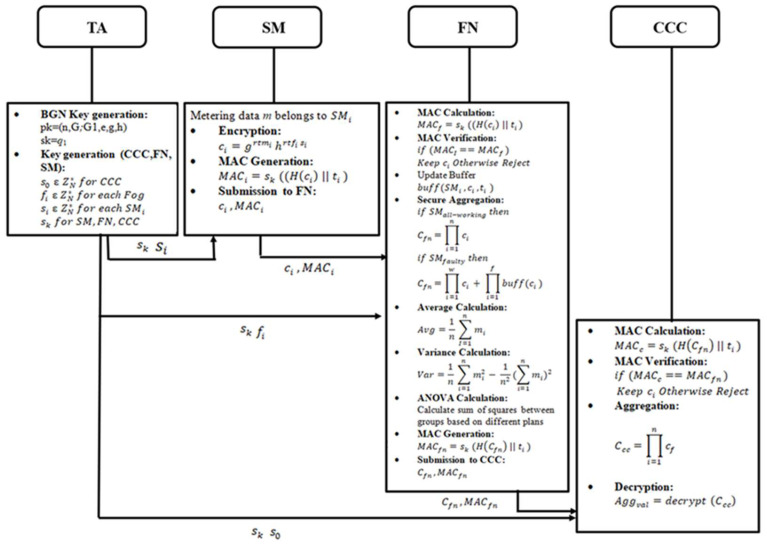
Block diagram of the proposed scheme.

Figure 4 illustrates the block diagram of the FESDAO scheme, which involves four primary entities: the Trusted Authority (TA), Smart Meters (SMs), Fog Nodes (FNs), and the Cloud Control Center (CCC).

The TA distributes secret parameters to the SMs, FN, and CCC. Each SM encrypts its metering data $m_{i}\;$ using its secret parameter, producing ciphertext $c_{i}.$ The SM also generates a MAC on ciphertext $c_{i}$ using its private key and concatenates it with a timestamp $t_{i}$. The SM shares the ciphertext $c_{i}$ and MAC ${MAC}_{i}$ both to the FN. Upon receipt, the FN verifies the MAC. If valid, the FN stores the data in a local buffer and aggregates the encrypted data from all SMs under its jurisdiction. In the case of failed SMs, the FN substitutes their missing readings with the most recent successful values stored in the buffer, ensuring fault-tolerant aggregation. The FN then generates its MAC ${MAC}_{fn}$ on aggregated data and forwarded it to the CCC. At the CCC, the MAC is verified, and the aggregated data from all FNs is combined and decrypted to compute the estimated electricity consumption values. This workflow ensures data confidentiality, integrity, and fault tolerance throughout the FESDAO scheme.

## 6. Security and Privacy Analysis

To demonstrate that the FESDAO scheme is privacy-preserving, supports analytical operations on encrypted data, and is secure under the defined threat model, we evaluated it through the following theorems, and it is shown that the proposed scheme preserves the data privacy of metering data, ensures source authentication, fault tolerance, and prevents FDI and replay attacks.

**Theorem** **1.** *The security of SM metering data is secured.*

**Proof.**  Suppose an adversary A eavesdrops on the communication while metering data is being transmitted from an SM to the FN. In this case, the adversary can observe the ciphertext $c_{i}$ of any SM. In the FESDAO scheme, the metering data is encrypted using the mBGN cryptosystem,

$$c_{i}=E(m_{i}){=g}^{{rtm}_{i}}h^{rts_{i}f_{i}}$$

To decrypt the ciphertext, the adversary would require the private key components held by the SM and FN, which are not available to it. In the above calculation r is also randomly chosen by CCC during the start of data aggregation, which is not known to the adversary. Therefore, the adversary cannot recover individual SM data, and its privacy is secured against indistinguishable under chosen-plaintext attack (IND-CPA). □

**Theorem** **2.** *If the set of SMs for a specific FN is compromised, still other* ${SM}_{i}$ *data is protected.*

**Proof.**  If adversary A, through any means, compromised some SMs and obtained their secret parameter $s_{i}$, It is easy to reveal those SMs private data. In the FESDAO scheme, secret parameter $s_{i}$ is randomly generated, and it has no relation with other SMs secret parameters. It means that if some SM secret parameters are known to the adversary, it is still not possible for it to know the metering data of other SMs. The condition below can reveal the aggregated data only if the private key of mBGN ($q_{1})$, the secret parameter value of FN ($f_{i}$) and secret parameters of all SMs ($s_{i}$) is known to adversary A.

$$f_{i}.(s_{1}+s_{2}+\ldots s_{n})=1\;mod\;q_{1}.$$

Let each ${SM}_{i}$ computes a partial value $D_{i}$ = $c^{s_{i}}$, (This is required for share-based decryption: each share is an exponent applied to the ciphertext). All $D_{i}$ are sent (or aggregated) to the combiner (FN). The combiner forms the product and applies its combining coefficient:

$$D=\prod_{i=1}^{n}D_{i}=\prod_{i=1}^{n}c^{s_{i}}=C^{(s_{1}+s_{2}+\ldots s_{n}).}$$

$$f_{i}.(s_{1}+s_{2}+\ldots s_{n})=1\;mod\;q_{1}$$

FN raises D to the power $f_{i}$:

$$D^{f_{i}}={C^{\left(s_{1}+s_{2}+\ldots s_{n}\right)}}^{f_{i}}=c^{f_{i}(s_{1}+s_{2}+\ldots s_{n})}\equiv c^{1modq_{1}}$$

Because $f_{i}$.($s_{1}+s_{2}+\ldots s_{n}$) $=1\;mod\;q_{1}$ ≡ $c^{1modq_{1}}$ is working inside the subgroup of order $q_{1}$ this recovers c (or the needed representative) and lets the holder of the remaining private key material complete decryption. From this calculation, it is evident that the FN is unable to recover or infer the individual readings of any SM, ensuring data confidentiality. □

**Theorem** **3.** *If FN is compromised, it cannot reveal the individual user’s data.*

**Proof.**  If the FN is compromised due to an internal attack or malfunction, it can only expose encrypted aggregated data. Decryption can only be performed if data from all SMs associated with the FN is received. The decryption activity can only reveal total usage information at the FN level. Hence, the privacy of individual SM cannot be compromised by adversary. The FN performs decryption activity through the following Equations.

$$C_{I}\;={\left(g^{r\sum_{i}^{n}m_{i}}\;h^{r}\;\right)}^{tt^{-1}}$$

Divide both sides by $h^{r}$

$$\frac{C_{I}}{h^{r}}=g^{r\sum_{i}^{n}m_{i}}\frac{h^{r}}{h^{r}}$$

$$C_{I}h^{-r}=g^{r\sum_{i}^{n}m_{i}}$$

Using discrete log, FN computes

$$M_{sum}=\sum_{i}^{n}m_{i}$$

Similarly, if a strong adversary A compromises the CCC, they may obtain the aggregated consumption value M. However, since the CCC lacks the capability to decrypt individual users’ consumption data, the adversary cannot infer any specific user’s reading from the aggregate. Therefore, even though the adversary has successfully penetrated the CC, the individual privacy of users remains preserved. □

**Theorem** **4.** *An eavesdropper through a replay attack cannot change the state of aggregated data.*

**Proof.**  Because all data packets sent from SM to FN are time-stamped, the timestamp (TS) of a data packet can be checked; if the timestamp (TS) is for an earlier period, the data packet will be rejected. A timestamp can be found in both the metering data $c_{i}$ and the MAC $MAC_{I}$.

$$c_{i}=E\;(m_{i}){=g}^{{rtm}_{i}}\;h^{rt}$$

□

**Theorem** **5.** *An attacker cannot impersonate the FN.*

**Proof.**  If an adversary tries to send some fake data packets by hiding the actual meter’s identity, it will be identified through the MAC authentication mechanism. If MAC and identity are verified at the FN level, its data packets will be accepted; otherwise, they will be rejected. Hence, the integrity of the data is preserved.

$${MAC}_{v}=s_{k}\left(H\left(c_{i}\right)\;||\;t_{i\;}\right)$$

$${if\;({MAC}_{v}=MAC}_{i\;})\;Keep\;c_{i}\;Otherwise\;Reject$$

□

## 7. Performance Evaluation

This section presents the implementation results of the proposed secure aggregation scheme in fog-enabled SG architecture. We have evaluated the performance as below: To evaluate the performance, we have analyzed the aggregation cost in the presence of colluding parties and compared the results with existing schemes [2,12]. Implementation parameters are mentioned in Table 3.

- Aggregation costs at the FN and CCC level, as shown in Table 4.
- Calculate the proposed scheme cost of encryption, MAC generation, MAC verification, and decryption, as shown in Table 5 and Table 6. The encryption cost is compared with [2,12].
- Communication cost comparison is given in Table 7.
- Comparison of security properties with existing schemes, as shown in Table 8.
- Computation cost of three data analytical operations (average, variance, ANOVA).

The FESDAO scheme is implemented using Java (JDK 8.0) with the Java Cryptographic Extension (JCE). Experiments are conducted on a system with an 11th Gen Intel Core i7-1165G7 Quad-Core CPU (Intel Corporation, Santa Clara, CA, USA, 2.8 GHz, 8 threads), 16 GB DDR4 RAM (SK Hynix Inc., Seoul, Republic of Korea; 2400 MHz and 3200 MHz modules), running Microsoft Windows 11 Enterprise 64-bit (Microsoft Corporation, Redmond, WA, USA). The proposed research leverages the publicly available Individual Household Electric Power Consumption dataset from Sceaux, Paris, France, hosted by the UCI Machine Learning Repository [31]. This dataset contains more than 2 million records collected between December 2006 and December 2010 and includes attributes such as SM identifiers, consumption values, and per-minute reporting intervals.

For key generation in the mBGN scheme, two large primes $q_{1}\;\mathrm{and}\;q_{2}$, each of 512 bits, are used. Similarly, 256-bit keys are employed for MAC generation. The initial parameter settings are summarized in Table 3. 

**Table 3:** Implementation parameters.

Parameter	Value Used
$q_{1}\;$	512 bits
$q_{2}$	512 bits
Hash Algorithm	256 bits

In the FESDAO scheme, there are n FNs, each of which collects consumption data from its connected SMs. Aggregation is performed at the FN level, where the collected consumption data is processed. The aggregation cost is calculated for three FNs (${fn}_{1}$, ${fn}_{2}$ and ${fn}_{3}$) and is presented in Table 4. The scheme can be easily extended to support additional FNs as needed, thereby ensuring scalability and accommodating extra load.

**Table 4:** Aggregation cost at fog nodes.

BGN based Aggregation Cost							
$Fn_{1}$ Level		$Fn_{2}$ Level		$Fn_{3}$ Level		CCC	
No# of SMs	Time (ms)	No# of SMs	Time (ms)	No# of SMs	Time (ms)	Total SM	Time (ms)
100	12	100	11	100	12	300	35
200	30	200	34	200	32	600	98
400	84	300	40	300	43	1000	167
500	101	400	87	500	81	1400	269
1000	166	500	103	500	104	2000	373

Table 5 presents the costs associated with encryption, MAC generation, MAC verification, and ciphertext storage at the FN. The evaluation of the FESDAO scheme is carried out with respect to computation, communication, and fault-tolerance factors.

**Table 5:** FESDAO cryptographic operation computation cost.

Level	Encryption	MAC-Generation	MAC-Verification	Decryption
$SM_{i}$	90 per SM	1 per SM	-	-
$FN_{i}$	-	1 per FN	2 per SM	34.1 per 100 SMs
CCC	-	-	2 per FN	2 per 3 FNs

### 7.1. Computation Cost

The computational cost of the proposed FESDAO scheme is evaluated based on the time required for encryption at the SM, MAC generation, aggregation, decryption, and the calculation of statistical functions at the FN and CCC. At the SM level, most operations consist of encryption using the mBGN private key and MAC generation on the encrypted data for authentication purposes. At the FN level, the main tasks include MAC verification, aggregation, statistical computations, and decryption. The FN also performs MAC generation and encryption when submitting aggregated results to the CCC. At the CCC, the aggregated data received from each FN undergoes MAC verification and decryption to determine the overall electricity consumption.

**Figure 5:**
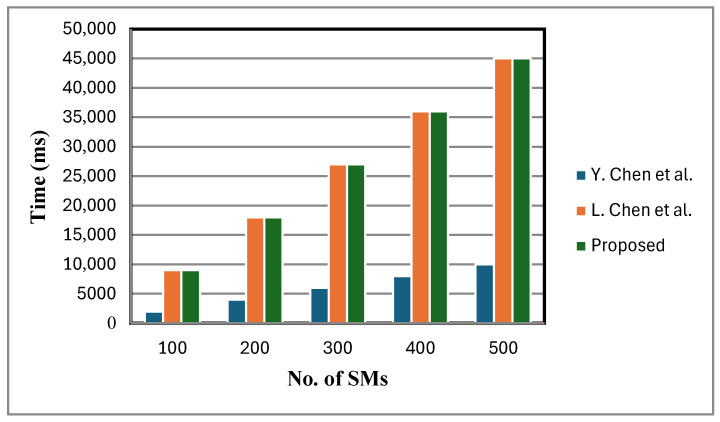
Encryption cost comparison [2,12].

Table 4 reports the aggregation cost at each FN and the total aggregation cost at the CCC. In Table 5, encryption cost comparison is given. The FESDAO scheme is compared with existing approaches that support statistical operations on ciphertext data during aggregation [2,12]. The encryption cost, calculated using Algorithm 2 and presented in Figure 5, shows that FESDAO and [2] have identical encryption costs, both being higher than [12]. This is because FESDAO and [2] use the BGN cryptosystem for encryption, whereas [12] employs Paillier encryption. The BGN scheme, which relies on bilinear maps and two cyclic groups, is computationally more expensive than Paillier. However, its key advantage lies in supporting both additive and multiplicative operations on ciphertext, while Paillier only supports additive operations. This makes BGN particularly useful for more complex statistical computations. 

**Table 6:** Encryption cost at the SM level.

No. of SM	L. Chen et al. [2]	Y. Chen et al. [12]	Proposed
100	9000	2000	9000
200	18,000	4000	18,000
300	27,000	6000	27,000
400	36,000	8000	36,000
500	45,000	10,000	45,000

**Figure 6:**
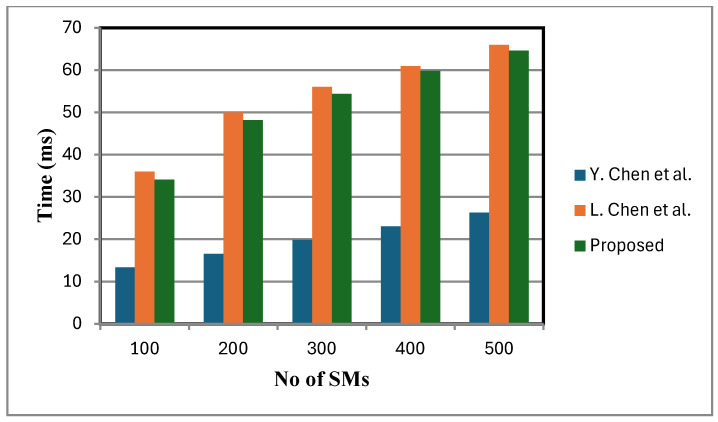
Decryption cost comparison [2,12].

**Figure 7:**
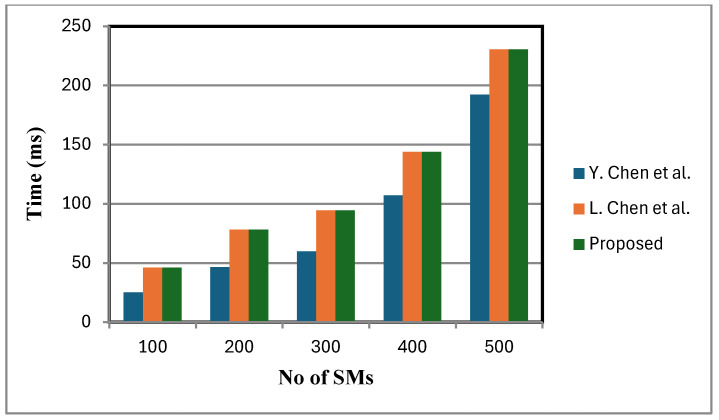
Average calculation comparison [2,12].

**Figure 8:**
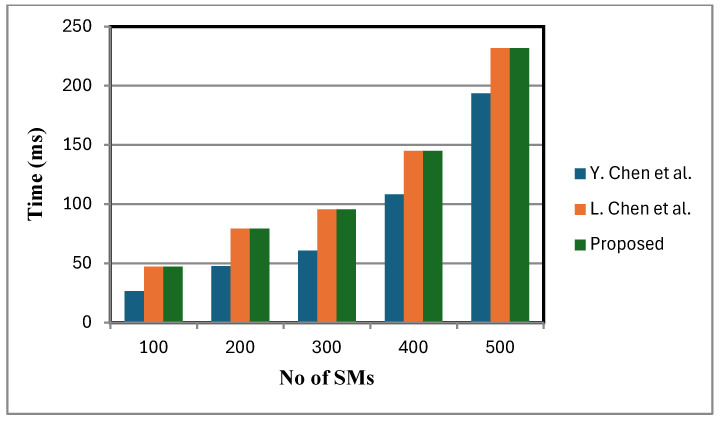
Variance calculation comparison [2,12].

**Figure 9:**
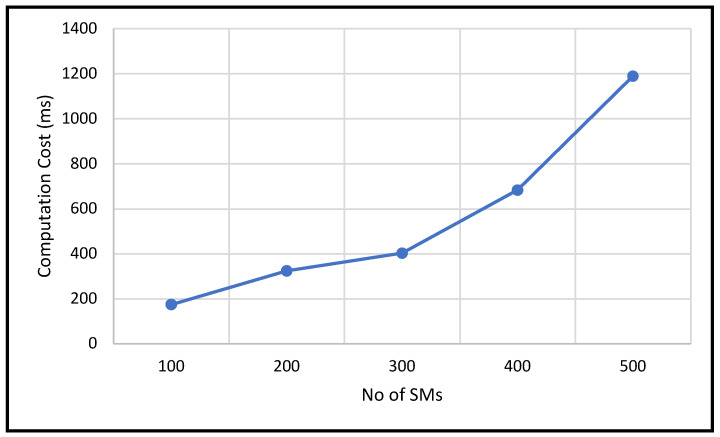
ANOVA computation time.

Figure 6 shows that the decryption cost of the proposed scheme is lower compared to that of [2]. This is because a small, fixed number of faulty sensor nodes are considered. By contrast, the schemes in [2,12] lack fault tolerance and do not support statistical operations at the FN. When meters fail, these schemes either ignore their data or adjust dummy inputs, leading to reduced accuracy in statistical calculations. In certain cases, the TA is additionally consulted to verify the status of failed meters, introducing communication overhead and delaying aggregation and decryption. While this may be manageable with a small number of failed meters, in large-scale failure scenarios it can lead to severely distorted analytical outcomes, under-mining the objectives of statistical analysis and reducing the overall efficiency of the smart grid. Figure 7, Figure 8 and Figure 9 present the computation cost, which is derived from the aggregation cost, decryption cost, and computation operations at the fog node. We assume no faulty SMs in Figure 7, Figure 8 and Figure 9. In the proposed scheme, both aggregation and decryption costs are based on the BGN cryptosystem.

Decryption is performed only after the aggregation process, with the aggregation cost excluding decryption cost. Figure 7 depicts variance calculation scales linearly with the number of inputs. Since ANOVA involves similar computational expressions, with data partitioned into groups, its computation time is also expected to grow linearly.

In this experiment, to demonstrate how ANOVA is computed, we used a practical example involving three different electricity tariff plans. The ANOVA analysis was conducted on sample data collected from SM connected to a FN, corresponding to tariffs labeled A, B, and C. On the *x*-axis, we plotted the total number of observations received at the FN, with values of 100, 200, 300, 400, and 500. For instance, the point at 100 on the *x*-axis indicates that the combined number of observations from all three tariff plans is 100. The *y*-axis represents the computation cost measured in milliseconds. The graph shows in Figure 9 that as the number of observations increases, the computation time at the FN grows roughly in a linear fashion.

### 7.2. Communication Cost

In the FESDAO scheme, the communication cost is evaluated by comparing the size and number of message exchanges between the SM and the FN, as well as between the FN and the CCC. The comparative results of all three schemes are summarized in Table 7.

**Table 7:** Communication cost from SM to FN.

Scheme	Security Parameter	Cipher Text Size	Time Stamp	MAC Generation	SM ID# Size	Per SM Total	For all SMs
**Proposed**	512	1024	32	256	32	1344	1344 × N
**L. Chen et al. [2]**	512	-	-	-	-	1024	1024 × N
**Y. Chen et al. [12]**	1024	2048	32	256	32	2368	2368 × N

N denotes the number of smart meters (SMs).

In the FESDAO scheme security parameter n is considered as 512 bits, and the size of the ciphertext $c_{i}$ is 1024 bits. For source authentication, HMAC with SHA-256 is employed, producing a 256-bit tag. To mitigate replay attacks, a 32-bit timestamp and a 32-bit SM identification number are included. Consequently, the total communication cost for a single SM is calculated as: 1024 bits (ciphertext) + 256 bits (MAC) + 32 bits (timestamp) + 32 bits (ID) = 1344 bits. For all N SMs, the aggregate upstream communication cost is therefore 1344 × N bits. In contrast, the communication cost in scheme [2] is reported as 1024 bits, as the authors only consider the ciphertext size and omit authentication and timestamp overheads. The communication cost of [12] from SM to FN is 3268 bits, and FN to CCC it is also 2368 bits. For Pailler system, the bit length of k is considered as 1024 bits, and $n_{2}$ as 2048 bits. For signature generation SHA-256 is used. Timestamp value of 32 bit along with the 32 bits identification number of SM is added to make a complete packet of 2368 bits. However, at FN, the communication cost varies across all three schemes. The FESDAO scheme adjusts metering data to reflect aggregated values for failed meters, while in schemes [2,12], it is ignored. Figure 9 represents communication cost from SM to FN.

**Figure 10:**
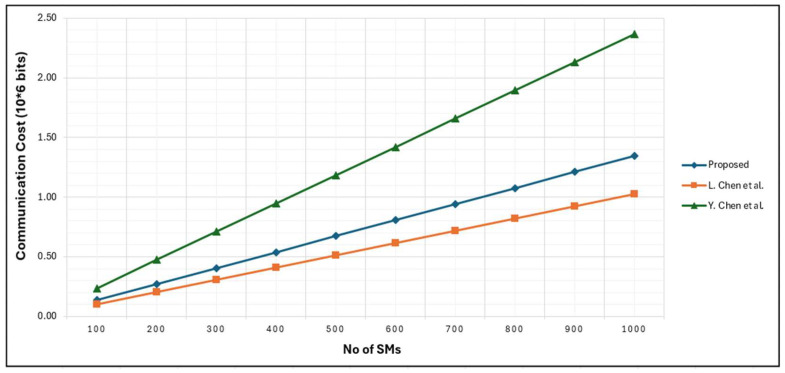
Communication Cost from SM to FN [2,12].

The FESDAO fault tolerance mechanism adjusts metering data to reflect values for failed meters, while in schemes [2,12], it is ignored. These schemes not only increase latency but also lead to inaccurate outcomes in data analytical operations. Figure 9 represents communication cost from SM to FN while assuming no faulty meter. The schemes [2,12] require an additional round of communication during data aggregation to verify SM data via the trusted authority (TA). The comparative communication costs from SM to FN are summarized in Figure 10.

### 7.3. Fault Tolerance

Local buffers are used in the proposed scheme to store metering data prior to aggregation. If no data is received for faulty SMs, their last successful reading will be adjusted to complete the aggregation process. In comparison to existing state-of-the-art schemes [2,12], where faulty SMs are ignored, and the fault tolerance scenario is not considered during data aggregation. If missing SMs data is ignored, the final decrypted usage information is inaccurate and cannot be used for forecasting or demand-response requirements. Compared to the schemes [2,12], the proposed scheme has much practical support for fault tolerance. The placement of real values enables the aggregation process to be completed efficiently, without requiring involvement from other parties. This approach ensures that, even if up to 50% of smart meters are faulty, latency remains largely unaffected, though there may be a slight reduction in data analytics accuracy. As the proportion of faulty meters increases further, the accuracy of analytics will decrease, since substituted values rely on prior periods.

The proposed scheme improves accuracy through two-way communication between faulty SMs and FNs. When a failed SM becomes available, the FN notifies it that the last reading for the missing period has been adjusted. This allows the SM to update its metering data for future submission cycles. Additionally, SMs that fail to submit data for more than three consecutive periods will be suspended, and their cases will be referred to the trusted authority (TA). The buffer requirement to store the last successful readings is minimal, and fog-based SGs can easily handle it due to an abundance of resources.

### 7.4. Random Meters Addition and Removal

The proposed scheme has no dependency on the addition or removal of SMs. In case of adding a new SM, secret share $s_{i}$ needs to be generated and shared with the new ${sm}_{i}.$ The TA will also update the condition for the new secret key as $f_{i}$.($s_{1}+\;s_{2}+\;\ldots s_{n}$) $=1\;mod\;q_{1}$. Similarly, in the case of removal, the secret key needs to be removed from the Equation $f_{i}$.($s_{1}+\;s_{2}+\;\ldots s_{n}$) $=1\;mod\;q_{1}$. The fault-tolerant algorithm is independent of meter addition or removal.

Table 8 presents the security properties addressed by the state-of-the-art and proposed scheme. The proposed scheme supports two extra properties: statistical operations support at the FN and CCC levels and Fault tolerance during secure aggregation. Privacy is achieved through homomorphic encryption; integrity and authentication are achieved through MAC tag generation on the encrypted ciphertext. False data injection attacks are prevented through timestamps in every message. Based on the timestamp, old packets are rejected.

## 8. Conclusions

This paper presents a fog-enabled secure data aggregation (FESDAO) scheme with integrated fault tolerance, designed to strengthen the reliability and efficiency of smart grid data analytics. The scheme highlights the critical role of statistical analysis in uncovering patterns and insights from consumption data, while simultaneously addressing privacy and security requirements that are essential for maximizing the value of data-driven decision-making in smart grids.

The proposed FESDAO framework leverages fog computing to extend analytical capabilities for fog node, and cloud control center layers. It ensures key security properties, including privacy, authentication, and integrity, while maintaining fault tolerance during secure aggregation. Unlike existing approaches that either neglect fault tolerance or treat failed smart meters as null inputs, FESDAO incorporates valid historical data in aggregation without requiring additional communication with smart meters or the trusted authority.

To provide robust protection against false data injection attacks, the scheme employs modified BGN and other mechanisms. Furthermore, it enables secure execution of advanced analytical operations such as average, variance, and ANOVA directly on encrypted data at both fog nodes and the cloud control center. This capability supports real-time analysis of energy consumption patterns, thereby facilitating the design of adaptive tariff strategies and empowering consumers to make informed decisions regarding their energy usage.

In future work, we plan to extend the FESDAO framework to support more advanced statistical functions and analytics on encrypted data. We also aim to enable statistical function calculations at the SM level, where appliances within individual households can submit their data to the local SM for aggregation and analysis. This will open opportunities for fine-grained energy usage insights, and personalized feedback for consumers. Furthermore, we plan to simulate the scheme in more practical and large-scale environments to evaluate its performance and scalability across a larger number of smart meters, ensuring its applicability to real-world smart grid deployments.

## Figures and Tables

**Table 1:** Tariff ANOVA example.

Group	$n_{j}$	$\sum x_{j}$	$\sum x_{j}^{2}$	${mean}_{j}$
Tariff-A	24	124.62	657.476	5.1925
Tariff-B	22	133.053	809.389	6.04877
Tariff-C	26	151.024	870.436	5.80862

**Table 8:** Comparison of security properties.

Properties	L. Chen et al. [2]	Y. Chen et al. [12]	Proposed Scheme
Privacy	✔	✔	✔
Integrity	✔	✔	✔
Authentication	✔	✔	✔
FDI	✘	✔	✔
Collusive Attack	✘	✘	✔
Basic Statistical Functions at FN	✘	✘	✔
Basic Functions at CCC Level	✔	✔	✔
ANOVA calculation at FN	✘	✘	✔
ANOVA calculation at CCC	✘	✘	✔
Fault Tolerance at FN	✘	✘	✔
Fault Tolerance at FN	✘	✘	✔

## Data Availability

Data are contained within the article.
